# The MYB transcription factor *CiMYB42* regulates limonoids biosynthesis in citrus

**DOI:** 10.1186/s12870-020-02475-4

**Published:** 2020-06-03

**Authors:** Pan Zhang, Xiaofeng Liu, Xin Xu, Fusheng Wang, Junhong Long, Wanxia Shen, Dong Jiang, Xiaochun Zhao

**Affiliations:** 1 Citrus Research Institute, Southwest University/Chinese Academy of Agricultural Sciences, Beibei, Chongqing, 400712 China; 2 National Citrus Engineering Research Center, Beibei, Chongqing, 400712 China

**Keywords:** Limonoid biosynthesis, R2R3MYB, *CiMYB42*, *CiOSC*, Triterpenoid

## Abstract

**Background:**

Limonoids are major bioactive compounds that are produced by the triterpenoid metabolic pathway. The detailed biochemical process of limonoid biosynthesis and the mechanism of its molecular regulation remain elusive. The identification of transcription factors that regulate limonoid biosynthetic pathways is very important for understanding the underlying regulatory mechanisms. This information could also provide tools for manipulating biosynthesis genes to modulate limonoid production.

**Results:**

In this study, the *CiMYB42* transcription factor was isolated to identify its role in limonoid biosynthesis. Multiple alignment analysis and phylogenetic analysis demonstrated that *CiMYB42* is a typical *R2R3MYB* transcription factor that shares high similarity of its amino acid sequence with *AtMYB42*. Limonoids contents were higher in *Citrus sinensis* and *Citrus grandis* than in other species. Limonoid accumulation during leaf development also showed diverse trends in different genotypes. The expression of *CiMYB42* was significantly related to the limonoid content and the expression of *CiOSC* in some citrus accessions. The overexpression of *CiMYB42* in sweet orange resulted in significant accumulation of limonin, whereas the downregulation of *CiMYB42* by RNAi resulted in a dwarf phenotype and less nomilin accumulation. Furthermore, the results of a yeast one-hybrid assay and EMSA indicated that *CiMYB42* binds exclusively to the TTGTTG sequence (type II MYB core) in the promoter of *CiOSC*. Together, these results suggest that *CiMYB42* positively regulates limonoid biosynthesis by regulating the expression of *CiOSC* by binding to the TTGTTG sequence (type II MYB core) of its promoter.

**Conclusions:**

*CiMYB42* is an important transcription activator involved in limonoid biosynthesis that regulates the expression of *CiOSC* by binding to the TTGTTG sequence (type II MYB core).

## Background

Citrus is one of the most important fruit crops in the world. Citrus produces diverse secondary metabolites, including limonoids. Limonoids possess extensive biological and pharmacological activities [1], such as antioxidant [2] and insect antifeedant [3, 4] as well as antibacterial [5, 6], anticancer [7–9], antiviral [10, 11], and anti-inflammatory [12] activities. The production of limonoids varies in different citrus species, organs and tissues, and developmental stages [13–15].

Limonoids are tetracyclic triterpene compounds that are synthesized from isopentenyl diphosphate (IPP) and dimethylallyl diphosphate (DMAPP) via the mevalonate (MVA) pathway and methylerythritol phosphate (MEP) pathway, respectively [16, 17]. The condensation of IPP and DMAPP forms C15 farnesyl diphosphate (FPP), which is further transformed into the linear C30 triterpenoid precursor squalene catalysed by squalene synthase (SQS) in a head-to-head condensation reaction. Subsequently, squalene epoxidase (SQE) oxidizes squalene to form 2,3-oxidosqualene, which undergoes cyclization mediated by specific oxidosqualene cyclases (OSCs) to form diverse triterpenoid skeletons [18]. A schematic diagram of limonoid biosynthesis is shown in Fig. S1. Squalene is the first precursor of triterpenoids such as limonoids, sterols, and brassinosteroids. SQS plays an important regulatory role in triterpenoid biosynthesis because it is located at a key branch point and acts as a switch [19]. SQE and OSC are the key rate-limiting enzymes in triterpenoid biosynthesis, catalysing the first oxygenation and cyclization steps, respectively [20, 21]. Strategies for altering triterpenoid production by manipulating genes that encode triterpenoid pathway enzymes have been reported [22–25]. Transcription factors (TFs) present great potential for improving the production of secondary metabolites by activating or repressing structural genes in metabolic pathways by binding to their promoter regions [26]. Hence, they are ideal targets for genetically manipulating the production of triterpenoids.

None of the TFs found in citrus have been reported to regulate triterpenoid production, but several TFs from other plants involved in triterpenoid biosynthesis have been identified. Shang et al. [27] reported that the *Bl* (bitter leaf) and *Bt* (bitter fruit) bHLH TFs regulate the biosynthesis of cucurbitacin C by binding to the promoter of *Bi* (a member of *OSC*) in cucumber (*Cucumis sativus*). *TSAR1* (*TRITERPENE SAPONIN BIOSYNTHESIS ACTIVATING REGULATOR1*) and *TSAR2* are two homologous jasmonate-inducible bHLH transcription factors that directly influence triterpene saponin biosynthesis by interacting with the promoters of *HMGR1* (*3-HYDROXY-3-METHYLGLUTARYLCOENZYME A REDUCTASE1*) and *MAKIBISHI1* in *Medicago truncatula* [28]. The liquorice (*Glycyrrhiza uralensis*) bHLH TF *GubHLH3* positively regulates the expression of triterpenoid saponin biosynthetic genes [29]. Recently, *WsWRKY1* of *Withania somnifera* was found to directly regulate the triterpenoid pathway by binding to W-box sequences in the promoters of *SQS* and *SQE* [30]. In addition, MYB TFs are crucial regulators that participate in plant terpenoid metabolism. The overexpression of grapevine (*Vitis vinifera*) *VvMYB5b* in tomato induced interesting effects, including the downregulation of phenylpropanoid metabolism and beta-amyrin and upregulation of beta-carotene was up regulated [31]. *P. taeda PtMYB14* is also related to terpenoid biosynthesis [32, 33]. Both *PtMYB14* and *VvMYB5b* are members of *R2R3-MYB*s, which are likely to regulate terpenoid biosynthesis. Recently, another *R2R3-MYB* member, *SmMYB36* from *Salvia miltiorrhiza* Bunge, has been reported to promote the accumulation of diterpenoids (tanshinone) [34].

*R2R3MYB* transcription factors are one of the largest families of plant TFs. The members of this family extensively participate in terpenoid biosynthesis, not only that of triterpenoids but also those of other terpenoids. For example, spearmint (*Mentha spicata*) *MsMYB* can bind to the *cis-elements* of *MsGPPS. LSU* and suppress monoterpene biosynthesis [35]. *Artemisia annua AaMYB1* acts as an activator in diterpene metabolism (artemisinin, AN) [36]. Despite the identification of *R2R3MYB*s in plant terpenoid biosynthesis, their roles in triterpenoid metabolism are still poorly understood, especially in limonoid biosynthesis.

In our previous study, ciclev10021695m, an MYB family TF, was revealed to be related to the biosynthesis of limonoids by RNA-seq analysis [20]. In this study, the role of *CiMYB42* was investigated to elucidate the regulatory mechanism of *CiMYB42* in the biosynthesis of limonoids in citrus.

## Results

### Characteristics of *CiMYB42*

**Fig. 1:**
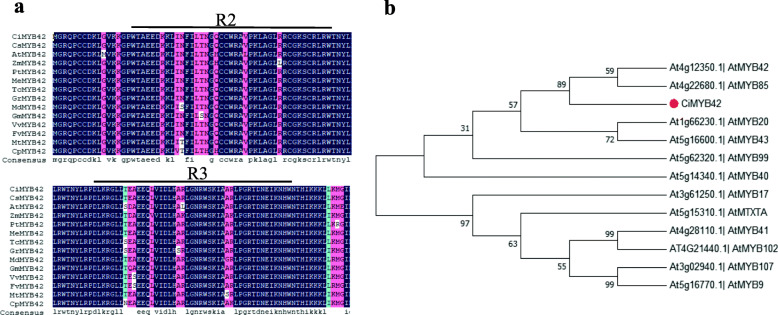
Amino acid sequence alignment and phylogenetic analysis. (a) Alignment of CiMYB42 and its homologous sequences in other species. The amino acid sequences shown are: *C. clementina* (ciclev10021695m); *C. sinensis* (orange1.1g024441m); *Arabidopsis thaliana* (AT4G12350.1); *Zea mays* (GRMZM2G104551_T01); *Populus trichocarpa* (Potri.003G114100.1); *Manihot esculenta* (Manes.02G034300.1); *Theobroma cacao* (Thecc1EG016054t1); *Gossypium raimondii* (Gorai.003G168100.1); *Malus domestica* (MDP0000682032); *Glycine max* (Glyma.01G211500.1); *Vitis vinifera* (GSVIVT01004317001); *Fragaria vesca subsp. vesca* (mrna32268.1-v1.0-hybrid); *Medicago truncatula* (Medtr4g102380.1); *Carica papaya* (evm.model.supercontig_3.300). (b) Phylogenetic tree of CiMYB42 and R2R3MYB transcription factors from *Arabidopsis thaliana*. The phylogenetic tree was constructed using MEGA 7

The genomic sequence of ciclev10021695m was obtained from the *C. clementina* genomic database (https://phytozome.jgi.doe.gov/pz/portal.html#!info?alias=Org_Cclementina). It encodes 267 amino acids and has a theoretical isoelectric point and molecular weight of 5.14 and 30.21 kDa, respectively. Ciclev10021695m is an R2R3 MYB transcription factor because it contains a typical conserved R2R3 MYB domain. The amino acid sequence of ciclev10021695m shares high similarity with *AtMYB42* of *Arabidopsis* (55.86%), and it was accordingly designated *CiMYB42* (Fig. 1a & b).

Phylogenetic analysis of *CiMYB42* with other *R2R3-MYB* genes from *Arabidopsis* indicated that *CiMYB42* is closely related to *AtMYB42* and *AtMYB85*, which have been reported to be involved in plant secondary metabolism [37]. In addition, our previous work demonstrated that the expression level of *CiMYB42* is significantly related to limonoid content in pummelo seeds [20]. This suggested that *CiMYB42* could act as a regulatory factor in limonoid biosynthesis.

### Accumulation of limonoids during leaf development

**Fig. 2:**
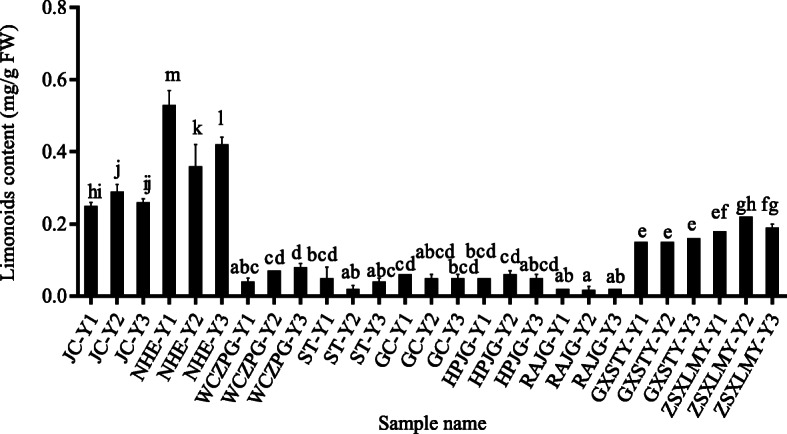
Limonoids content in different citrus varieties during leaf development. The abbreviations in figure are as follows: JC, Beibei 447 orange; NHE, Newhall navel orange; WCZPG, Wangcang Zoupigan mandarin; ST, Morita Unshu Mandarin; GC, Miyagawa Wase Mandarin; HPJG, Huapi kumquat; RAJG, Rongan kumquat; GXSTY, Guangxi Shatian pummelo; ZSXLMY, Early Siam pummelo; Y1, Y2 and Y3 are three leaf development stages, respectively

Leaf samples from nine different citrus accessions at three different developmental stages (Fig. S2) were used for the determination of limonoid (limonin and nomilin) contents in this study. The scientific names and abbreviations of nine citrus accessions were shown in Table 1. Limonoid contents varied among different accessions and leaf developmental stages (Fig. 2) and ranged from 0.02 mg/g FW to 0.53 mg/g FW in the nine citrus accessions. The highest limonoid content was 26 times greater than the lowest. Variation in limonoid contents among different citrus species was also observed. *C. sinensis* presented the highest limonoid content (0.25–0.53 mg/g FW), followed by *C. grandis* (0.15–0.22 mg/g FW). However, limonoid contents remained at a quite low level in *C. reticulata* and *F. classifolia*, especially in *F. classifolia.* This revealed that the genotype is one of the most important factors influencing limonoid content. In addition, several different patterns of limonoid accumulation during leaf developmental stages were found in different accessions (Fig. 2). Specifically, limonoid content first increased and then decreased throughout the period of leaf growth and maturation in JC and ZSXLMY but changed little. The limonoid content of NHE changed the most markedly and noticeably decreased in the Y2 period. In ST, it remained at a quite low level throughout the leaf growth period, and the trend of the changes was similar to that in NHE. In WCZPG, the limonoid content constantly increased during growth, reaching 0.04, 0.07 and 0.08 mg/g FW. The limonoid content showed no obvious change in the other varieties.

**Table 1:** Citrus samples used in this study

Name of species	Name of accessions	Abbreviation used in this study
*Citrus sinensis* (L.) Osbeck	Beibei 447 orange	JC
*Citrus sinensis* (L.) Osbeck	Newhall navel orange	NHE
*Citrus reticulata* Blanco	Wangcang Zoupigan mandarin	WCZPG
*Citrus reticulata* Blanco	Morita Unshu mandarin	ST
*Citrus reticulata* Blanco	Miyagawa Wase mandarin	GC
*Fortunella classifolia* Swingle	Huapi kumquat	HPJG
*Fortunella classifolia* Swingle	Rongan kumquat	RAJG
*Citrus grandis* (L.) Osbeck	Guangxi Shatianyou pummelo	GXSTY
*Citrus grandis* (L.) Osbeck	Early Siam pummelo	ZSXLMY

### Correlation between *CiMYB42* expression and limonoid content

**Fig. 3:**
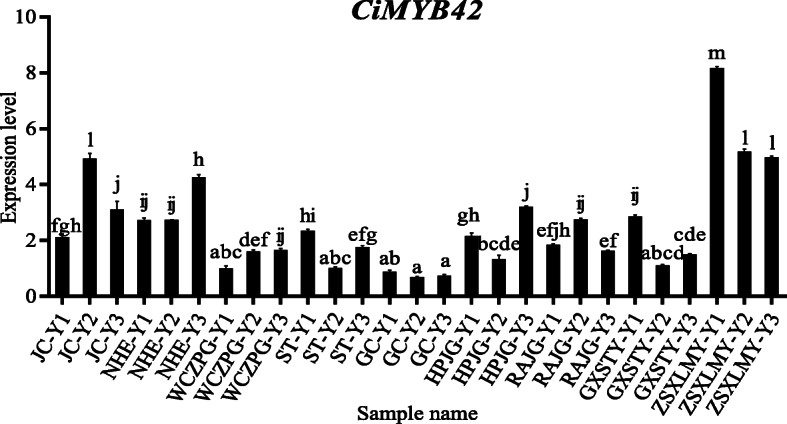
CiMYB42 expression level in different citrus accessions during leaf development. The abbreviations are: JC, Beibei 447 orange; NHE, Newhall navel orange; WCZPG, Wangcang Zoupigan mandarin; ST, Morita Unshu Mandarin; GC, Miyagawa Wase Mandarin; HPJG, Huapi kumquat; RAJG, Rongan kumquat; GXSTY, Guangxi Shatian pummelo; ZSXLMY, Early Siam pummelo; Y1, Y2 and Y3 are three leaf development stages, respectively

The relative expression of *CiMYB42* exhibited significant differences among the accessions and leaf developmental stages (Fig. 3). The highest expression level (8.17) was observed in ZSXLMY at the Y2 stage, whereas the lowest expression level (0.70) was found in the Y2 stage of GC, which presented 12-fold lower expression. The expression level of *CiMYB42* showed trends similar to those of limonoid content during leaf development. Significant positive correlations between the level of *CiMYB42* expression and limonoid content during leaf development were found in JC, WCZPG and GC (Table 2). However, some strong negative correlations were also observed (HPJG, GXSTY and ZSXLMY). Positive correlations between *CiMYB42* expression and limonoid content were found at all three leaf developmental stages among these accessions, especially in the Y3 stages (0.236, 0.639 and 0.66, respectively). Similar correlations were observed between limonoid content and *CiOSC* expression. However, the expression of *CiMYB42* presented a significant correlation with *CiOSC* during leaf development in most of the accessions, with the exception of GC. A close correlation was observed among the accessions in all three developmental stages (0.971, 0.824 and 0.81, respectively).

**Table 2:** Correlations between *CiMYB42* expression and limonoids content

Pearson Correlation Coefficient	JC	NHE	WCZPG	ST	GC	HPJG	RAJG	GXSTY	ZSXLMY
*CiMYB42*& Limonoids	0.78*	−0.155	0.835**	0.615	0.691*	−0.768*	−0.25	− 0.781*	−0.666*
*CiOSC*& Limonoids	0.737*	−0.321	0.885**	0.575	−0.736*	−0.701*	− 0.382	−0.359	− 0.703*
*CiMYB42*& *CiOSC*	0.943**	0.942**	0.689*	0.963**	−0.844**	0.855**	0.964**	0.708**	0.998**

* *P* < 0.05; ** *P* < 0.01

### Effect of *CiMYB42* on limonoid production in transgenic plants

**Fig. 4:**
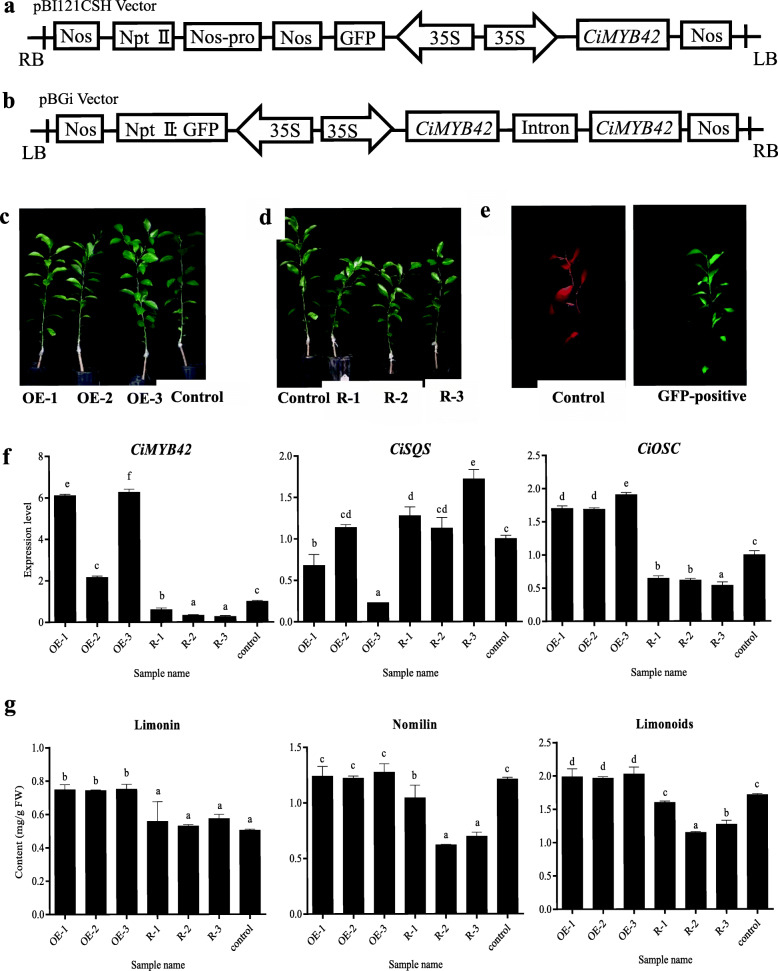
Analysis of CiMYB42 overexpression lines and RNAi lines. **a** A schematic diagram representing recombinant vector of overexpression; **b** A schematic diagram representing recombinant vector of RNAi; **c** Images of control and CiMYB42-overexpressing transgenic plants; **d** Images of control and CiMYB42-RNAi transgenic plants; **e** Images of GFP-fluorescent detection. GFP-positive is R-2 transgenic plants; **f** RT-qPCR analysis of CiMYB42, CiSQS, CiOSC expressions in overexpression and RNAi transgenic lines; **g** Limonin, nomilin and limonoids of transgenic plants

The overexpression and RNA interference knockdown of *CiMYB42* were conducted to further elucidate the role of *CiMYB42* in limonoid biosynthesis. The recombinant overexpression and RNA interference vectors are shown in Fig. 4a & Fig. 4b. Non-transgenic Wanjincheng orange (*C. sinensis* (L.) Osbeck) was grafted onto the rootstock used as the control. Three *CiMYB42*-overexpressing (*CiMYB42*-OE) lines and three *CiMYB42* RNA interference (*CiMYB42*-R) lines (Fig. 4c & Fig. 4d) were obtained via *Agrobacterium*-mediated transformation. Images of the control and GFP-positive cells under an ultraviolet lamp are shown in Fig. 4e. R-2 transgenic plants were used as an example to provide GFP-positive images. No morphological differences were observed between the control and overexpression lines, but the RNAi lines exhibited dwarfing and shorter internode characteristics (Fig. 4d). Subsequently, real-time qPCR was performed to evaluate the expression levels of *CiMYB42*, *CiSQS* and *CiOSC* in the transgenic lines and controls (Fig. 4f). *CiMYB42* expression was significantly higher in the overexpression lines than in the control, especially in overexpression lines 1 and 3, which showed approximately 6.6 times higher expression than the control. Three RNAi transgenic lines (R-1, R-2 and R-3) showed reduced levels of *CiMYB42* expression to different extents, exhibiting expression decreases of 39, 67 and 72%, respectively. The expression of *CiSQS* was not suppressed by silencing *CiMYB42* in RNAi plants and was not upregulated in the overexpression plants (Fig. 4f). The correlation between the expression of these two genes was significantly negative. In contrast, the expression of *CiOSC* was consistent with the expression of *CiMYB42* in transgenic plants.

The altered expression of *CiOSC* and *CiMYB42* altered limonoid accumulation in the overexpression and RNAi lines. The limonoid content increased by 16.08% in the overexpression lines and decreased by 22.09% in the RNAi lines (Fig. 4g). Notably, transgenic *CiMYB42* had different effects on limonin and nomilin contents. The limonin content was increased by 50% in the overexpression plants, but there was no significant difference between the RNAi lines and the control. In contrast, nomilin contents exhibited a great decline in the RNAi lines, particularly in line 2 (decreased by 48.76%). In the overexpression lines, the nomilin content only showed a slight increase (3.31%). Thus, the overexpression of *CiMYB42* mainly increased the limonin content, while *CiMYB42* RNAi mostly decreased the nomilin content.

Correlation analysis indicated that limonoid contents were significantly correlated with the expression of *CiMYB42* and *CiOSC* in the transgenic plants, with Pearson correlation coefficients of 0.824 (P < 0.05) and 0.931 (P < 0.01), respectively. However, the limonoid content was negatively correlated with the expression of *CiSQS*.

### *CiMYB42* regulates limonoid biosynthesis by binding to the promoter of *CiOSC*

**Fig. 5:**
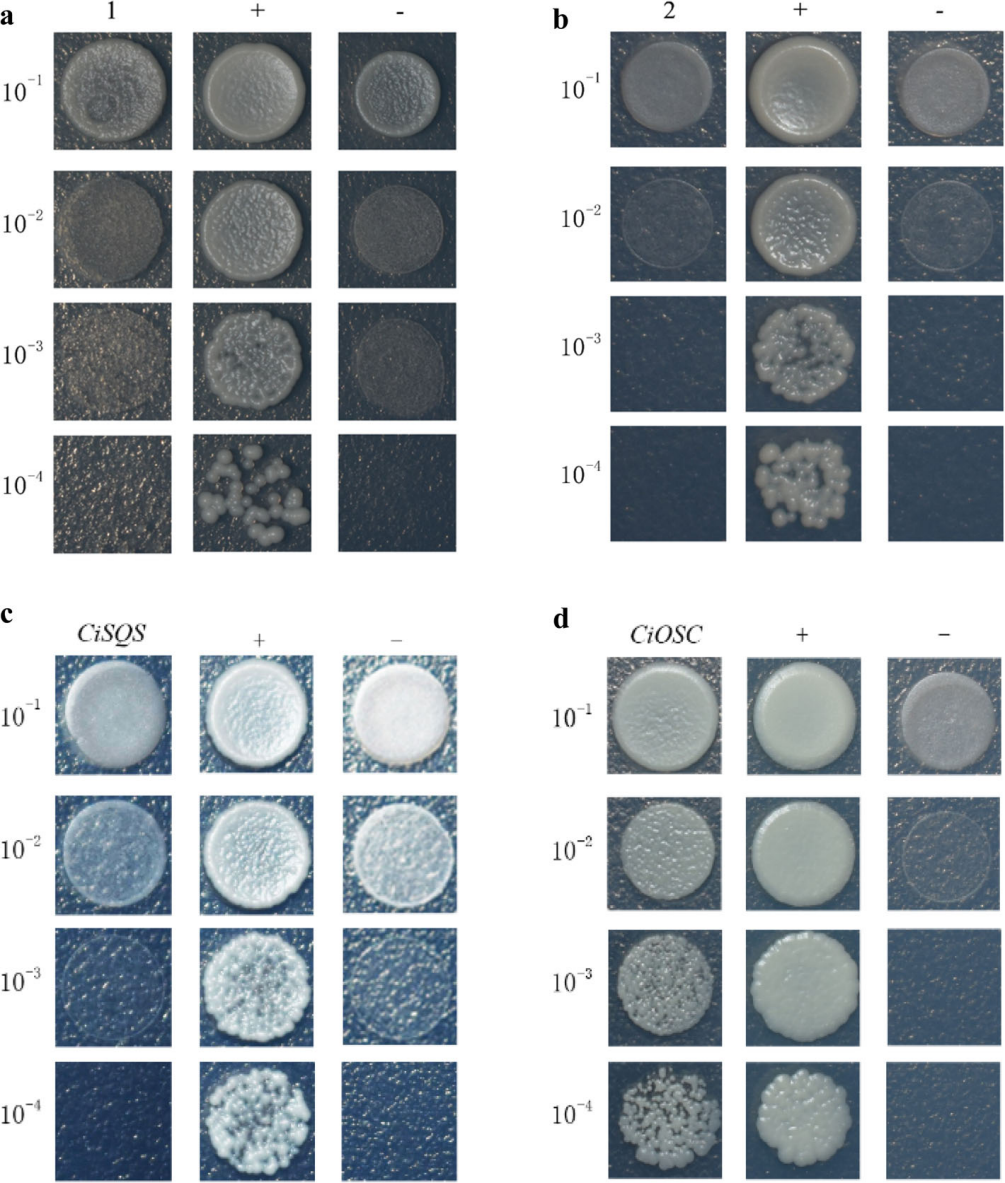
Identification of interaction between CiMYB42 and promoters of CiSQS and CiOSC with yeast one-hybrid assays. **a** Co-transformation of empty pGADT7 and pAbAi-SQS under the condition of 200 ng/ml AbA; **b** Co-transformation of empty pGADT7 and pAbAi-OSC under the condition of 500 ng/ml AbA; **c** The interaction between CiMYB42 and CiSQS under the condition of 200 ng/ml AbA; **d** The interaction between CiMYB42 and CiOSC under the condition of 500 ng/ml AbA; (+) Positive control: pGADT7-p53 + Y1H [pAbAi-p53]; (−) Negative control: pGADT7 + Y1H [pAbAi-p53]; (1) pGADT7 + Y1H [pAbAi-SQS]; (2) pGADT7 + Y1H [pAbAi-OSC]; the recombinant Y1H Gold yeast strain were selected by SD/ -leu medium containing optimal AbA concentrations; 10–1, 10–2, 10–3 and 10–4 are different dilution ratio

The significant correlations of gene expression between *CiMYB42*, *CiOSC*, and *CiSQS* suggested the possible interaction of *CiMYB42* with *CiOSC* or *CiSQS*. These TF binding *cis*-elements were identified in promoters of *CiOSC* and *CiSQS* by using PLANTCARE and PLACE online software (Fig. S3). The approximately 2 kb promoters of *CiOSC* and *CiSQS* contained several MYB cores and AC elements, which are required for MYB binding [38]. On the basis of these *cis*-element analyses, a Y1H assay was carried out to identify potential interactions between *CiMYB42* and the promoters of *CiOSC* and *CiSQS*. The minimal AbA inhibitory concentration of the bait vector was detected as shown in Fig. S4. In addition to the Y1H system control, the cotransformation of empty pGADT7 and pAbAi-SQS/OSC was also performed to reduce the false positive rate (Fig. 5a & b). The results indicated that *CiMYB42* directly and exclusively interacted with the *CiOSC* promoter under suppression by 500 ng/mL AbA (Fig. 5d). In contrast, there was no interaction between *CiMYB42* and the *CiSQS* promoter in yeast cells (Fig. 5c). This suggests that *CiMYB42* acts as an activator regulating the expression of *CiOSC*. These results indicated that *CiMYB42* could be one of the key factors involved in the biosynthesis of limonoids by regulating the expression of *CiOSC*.

### *CiMYB42* mediates *CiOSC* transactivation by binding to the type II MYB core *cis-element* in the *CiOSC* promoter

**Fig. 6:**
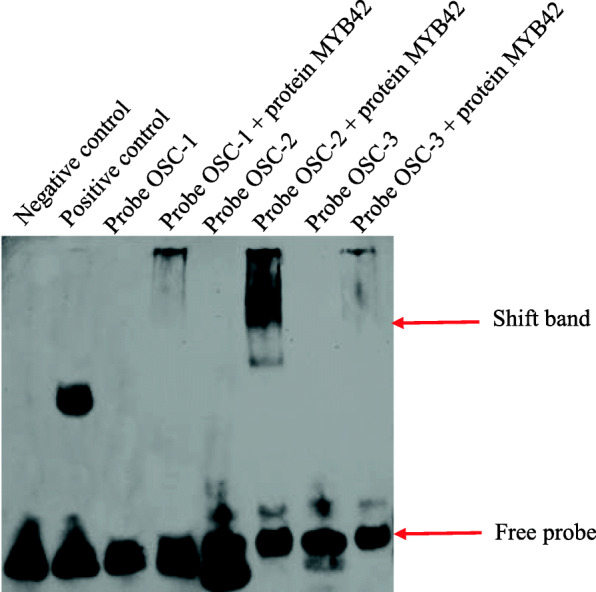
EMSA of CiMYB42 binding to the fragments of the CiOSC promoter. The recombinant protein of CiMYB42 fused with 6 × His tag was incubated with biotin-labeled probes and subjected to EMSA by polyacrylamide gel electrophoresis. Negative control: NF-κB probe (AGTTGAGGGGACTTTCCCAGGC); Positive control: NF-κB probe + nuclear protein of Hela cells. The gel image were cropped because original image includes some of other samples not related to this study

Many studied MYB proteins act via the recognition of the MYB core sequence (C/TNGTTG/A) and AC elements (ACCA/TAA/CT/C) in promoter regions [38, 39]. However, MYBs exhibit different affinities for these sequences. The pioneering work of Kelemen et al. [38] elucidated the preferential interaction of *AtMYB85* with the AC element and type II MYB core, especially for the type II MYB core. Based on the sequence similarity between *CiMYB42* and *AtMYB42* and *AtMYB85,* we deduced that *CiMYB42* may also interact with AC elements and the type II MYB core. Therefore, we performed electrophoretic mobility shift assays (EMSAs) with recombinant proteins and three biotin-labelled probes containing these *cis-element* sequences (Fig. 6). The expression and purification of the CiMYB42 protein are shown in Fig. S5. The original image of Figure S5 is shown in Figure S6. The original EMSA gel image is shown in Figure S7. As shown in Fig. 6, the C-terminally His-tagged CiMYB42 protein exhibited a strong affinity for the OSC-2 probe with the TTGTTG sequence but.

was not capable of binding to the OSC-1 (ACCAAAC, AC-element**)** and OSC-3 (TAACTA, type II MYB core) probes. These results showed that *CiMYB42* functions as an R2R3-type MYB transcriptional activator, which binds to the type II MYB core (TTGTTG) sequence in the *CiOSC* promoter and activates the transcription of the *CiOSC* gene in citrus.

## Discussion

In this study, the *CiMYB42* gene was identified as significantly affecting limonoid biosynthesis by regulating the expression of *CiOSC*. A previous report indicated that *CiMYB42* (ciclev10021695m) is an *R2R3MYB* gene located on scaffold S00271 of sweet orange (*C. sinensis*) and S3 of clementine (*C. reticulata*). Its expression can be induced by cold stress and ABA and JA treatments [40], but its function is unknown. In general, *R2R3MYB* transcription factors play important regulatory roles in terpenoid biosynthesis, especially the members in subgroups 4, 5, and 15 [33, 34]. *CiMYB42* was classified in subgroup 12 in a previous report [40]. Our study suggested that members of subgroup 12 may also be involved in the regulation of terpenoid biosynthesis.

Variations in limonoid contents in different species and developmental stages of citrus seeds and fruits have been reported [13, 15, 41, 42], but there has been less focus on limonoid accumulation during leaf development in different citrus species. In this study, limonoid contents were found to be significantly different among different leaf developmental stages in most of the examined accessions. The trends of limonoid accumulation during leaf development presented several different patterns depending on the genetic background. The expression of *CiMYB42* was not positively correlated with the limonoid contents of some accessions during leaf development (Table 1). However, similar correlations were observed between the expression of *CiOSC* and limonoid contents, possibly due to *CiOSC* indirectly participating in the limonoid biosynthetic pathway by regulating the synthesis of triterpene [43]. Furthermore, limonoid accumulation is affected not only by the biosynthesis of limonoids but also by factors such as the transportation and degradation of limonoids.

A reverse genetics approach was applied to determine the role of *CiMYB42* in limonoid biosynthesis. Some interesting phenotypes were identified in *CiMYB42*-silenced citrus plants (Fig. 4d). RNAi knockdown of *CiMYB42* resulted in stunted growth and shorter internodes of the plants exhibiting a significant decrease in limonoid contents. The phenomena of morphogenetic inhibition and reduced limonoid production resulting from the silencing of *CiMYB42* were similar to the findings of previous studies involving the downregulation/silencing of triterpenoid pathway genes and triterpenoid metabolism-related TFs such as *NtCAS1* [44], *AtHMGR1* [45] and *WsWRKY1* [30]. The decrease in the nomilin content induced by the RNAi knockdown of *CiMYB42* could be a result of the depletion of limonoid precursors, which are required for limonoid production [46]. The downregulation of *CiOSC* expression may also resulted in nomilin reduction. However, the small change in limonin production is likely due to a sufficient nomilin supply remaining for limonin synthesis despite the dramatic decrease in nomilin. A downregulation of *CiOSC* expression was induced by the RNAi knockdown of *CiMYB42* in citrus plants. However, *CiSQS* was slightly upregulated by *CiMYB42* RNAi. Such differential regulation of triterpenoid biosynthesis has recently been reported in *W. somnifera* (involving *WsWRKY1*) [30], in tomato (involving *GAME9*) [47], and in birch (*Betula platyphylla* Suk.; involving *BpbHLH9*) [48].

In contrast to *CiMYB42* RNAi, the overexpression of *CiMYB42* in sweet orange leaves increased the expression level of *CiOSC* and limonoid contents. Furthermore, the Y1H assay provided further support for the vital role of *CiMYB42* based on the finding that *CiMYB42* exclusively activated the promoter of *CiOSC*. These results are consistent with the conclusion that *CiMYB42* positively regulates limonoid biosynthesis. The overexpression of *CiMYB42* induced the accumulation of limonin rather than nomilin. This suggests that *CiMYB42* may have other targets. In addition to *CiOSC*, other downstream genes in the limonoid pathway may be induced by *CiMYB42*. One target gene may be regulated by several TFs; conversely, one TF may be involved in multiple biosynthetic processes [39, 49].

Previous reports showed that MYB TFs could bind to the MYB core sequence and AC elements [50, 51]. The EMSA results indicated that *CiMYB42* could specifically bind to the TTGTTG sequence of the *CiOSC* promoter (Fig. 6), suggesting that *CiMYB42* preferentially binds to the type II MYB core (TNGTTG/A), similar to *AtMYB85* [38]*.* Based on the results obtained for two close homologs of *CiMYB42*, *AtMYB42* and *AtMYB85* (Fig. 1b), suggest that *CiMYB42* may also regulate secondary cell wall biosynthesis. This is the first report elucidating the role of transcription factors in *Citrus limon*oid biosynthesis. Our contributions will provide a reference for understanding the regulatory mechanisms of R2R3MYB TFs in the triterpenoid biosynthetic pathway.

## Conclusions

In this study, we identified a novel regulatory factor, *CiMYB42*, that is involved in limonoid biosynthesis by binding to the type II MYB core (TNGTTG/A) sequence in the promoter of *CiOSC*. The results indicated that *CiMYB42* is a transcriptional activator in the limonoid metabolic network.

## Methods

### Plant materials and sampling

In April 2017, fresh healthy citrus leaves were collected at three different stages (Y1, Y2 and Y3) from different accessions in the National Citrus Germplasm Repository located in the Citrus Research Institute of the Chinese Academy of Agricultural Sciences (CRIC), in Beibei, Chongqing, China. Nine citrus accessions from four species were used for gene expression analysis and limonoid determination (Table 1). The leaf samples from the three developmental stages are shown in Fig. S2.

### Extraction and quantification of limonin and nomilin

The extraction and quantification of limonin and nomilin via HPLC were performed according to the method described by Sun et al. [13], and three biological replicates were performed. Because standard samples for most limonoid components are unavailable, only limonin and nomilin could be quantitatively analysed. Thus, the sum of nomilin and limonin was used to represent the limonoid content in this study.

### Bioinformation analysis of *CiMYB42*

The amino acid sequence of *CiMYB42* was obtained from the *C. clementina* genome database (https://phytozome.jgi.doe.gov/pz/portal.html#!info?alias=Org_Cclementina). The theoretical PI (isoelectric point) and MW (molecular weight) were predicted by EXPASY (https://web.expasy.org/compute_pi/). The simple modular architecture research tool (SMART) was used to confirm the domain sequence of *CiMYB42*. The protein sequences were subjected to BLAST searches against the Phytozome database to identify homologous sequences in other plant species. DNAMAN v.6.0 was used for multiple alignment analysis. The homologous sequences of *Arabidopsis* were used for the construction of the phylogenetic tree.

### DNA and RNA extraction, cDNA synthesis and relative expression analysis

Genomic DNA was extracted via the CTAB method, and RNA was extracted using the RNAprep Pure Plant Kit following the manufacturer’s instructions (Tiangen Biotech, Beijing, China). RNA (1 μg) was reverse transcribed into cDNA using the PrimeScript 1st Strand cDNA Synthesis Kit with gDNA Eraser (Perfect Real Time) (Takara Biomedical Technology, Beijing, China). The detection of gene expression was performed by real-time qPCR using 1× iTaq™ universal SYBR® Green Supermix (Bio-Rad). The primers used in these procedures are listed in supplementary Table. S1. Experiments were performed in three replications using the citrus *Actin* gene for normalization, and relative expression levels were calculated using the 2^-ΔΔCt^ method [52].

### Transformation and characterization of transgenic sweet orange

The CDS of *CiMYB42* was amplified and ligated into the *Sac* I*/Bam*H I sites of pBI121CSH to obtain the overexpression vector. For the construction of the RNAi vector, a 462 bp fragment was PCR amplified and integrated into the pGBi vector. These two expression vectors present enormous advantages in the visualization of exogenous gene transformation because GFP-positive samples are easily illuminated with an ultraviolet lamp (Fig. 4e). The recombinant overexpression and RNAi vectors were transformed into epicotyl explants of Wanjincheng orange (*Citrus sinensis* (L.) Osbeck) as previously reported [53]. The transformants were selected on MS medium containing 50 mg/ml kanamycin. The positive shoots were grafted onto rootstocks of two-year-old Ziyang Xiangcheng (*C. junos*) after the detection of GFP fluorescence and the PCR amplification of genomic DNA. The integration and expression of the *CiMYB42* gene in transformed shoots were further confirmed by RT-PCR analysis.

### Yeast one-hybrid (Y1H) assay

The full-length *CiMYB42* ORF was amplified and fused to the pGADT7 vector to create the prey for the assay. Sequences (2 kb) including the promoters of *CiSQS* (ciclev10028537m) and *CiOSC* (ciclev10010416m) were synthesized by Beijing Genomics Institute and inserted into the pAbAi vector as the bait. The primers used for vector construction are listed in Table. S1. Yeast one-hybrid (Y1H) assays were performed using the Matchmaker Gold Yeast One-Hybrid System (Clontech, USA). pAbAi-SQS and pAbAi-OSC were linearized by the BbsI enzyme, and the minimal inhibitory concentration of aureobasidin A (AbA) was detected on SD/−Ura medium. The interactions between *CiMYB42* and the promoters of *CiSQS* and *CiOSC* were subsequently tested by co-transformation of the linearized bait plasmid and prey plasmid into Y1H Gold yeast competent cells on SD/−leu medium with the optimal AbA concentration.

### Electronic mobility shift assay (EMSA)

A cDNA fragment of *CiMYB42* was amplified using gene-specific primers (Table S1) and inserted into the pMAL-C2X vector at the *Bam*HI/*Pst*I sites with a 6 × His tag. The recombinant pMAL-C2X-MYB42 plasmid was transformed into the *E. coli* Rosetta (DE3) strain. The pMAL-C2X-MYB42 protein was purified by the immobilized metal affinity chromatography method, and Ni Sepharose High Performance was used in this step according to the manufacturer’s instructions (GE Healthcare, USA). Oligonucleotide probes were synthesized and biotin labelled by Wuhan GeneCreate Biological Engineering Co., Ltd. The binding activity between the protein and probes was detected in an electronic mobility shift assay (EMSA). The CiMYB42-bound DNA fragments were separated from the unbound fragments by polyacrylamide gel electrophoresis according to the instructions of the Chemiluminescent EMSA Kit (Beyotime Biotechnology, China).

### Statistical analysis

Statistical analysis was performed using the SPSSV20.0 statistical package. Significant differences were subjected to Duncan’s test. *P* < 0.05 was considered significant. Correlation analysis was conducted via Pearson’s correlation analysis.

## Supplementary information

**Additional file 1: Table S1.** Primers and probes used in this study.

**Additional file 2: Figure S1.** Schematic diagram of limonoid biosynthesis. The abbreviations of the compounds and enzymes are as follows: IPP, isopentenyl diphosphate; DMAPP, dimethylallyl diphosphate; FPP, farnesyl diphosphate; FPPS, FPP synthase; SQS, squalene synthase; SQE, squalene epoxidase, OSC, oxidosuqlene cyclase; UDPG, UDP-glycosyltransferase; NG, nomilin-glucopyranoside; LG, limonin-glucopyranoside.

**Additional file 3: Figure S2.** Developmental stages of the leaf samples used in this study.

**Additional file 4: Figure S3.** The TF-binding cis-elements of the *CiSQS* and *CiOSC* promoters.

**Additional file 5: Figure S4.** The minimal AbA inhibitory concentration of the bait vector. (a) pAbAi-SQS; (b) pAbAi-OSC.

**Additional file 6: Figure S5.** Expression and purification of the CiMYB42 protein. (a) The recombinant CiMYB42 protein expressed in Rosetta (DE3); (b) The supernatant and deposition of the CiMYB42 protein were examined by SDS-PAGE after ultrasonication; (c) SDS-PAGE analysis of recombinant and purified CiMYB42 protein; Lane M: protein ladder (116.0/66.2/45.0/35.0/25.0/18.4/14.4 kD); Lane 1: uninduced Rosetta (DE3) carrying pMAL-C2X; Lane 2: Rosetta (DE3) carrying pMAL-C2X induced by 1 mM IPTG; Lane 3: supernatant of the CiMYB42 protein; Lane 4: precipitate of the CiMYB42 protein; Lane 5: purified CiMYB42 protein; Lane 6: purified CiMYB42 protein. The molecular weight of recombinant CiMYB42 protein is 72.21kD. The gel image were cropped because original image includes some of other samples not related to this study.

**Additional file 7 Figure S6.** The original gel image of Figure S5. (a) The original image of Figure S5a; (b) The original image of Figure S5b; (c) The original image of Figure S5c.

**Additional file 8 Figure S7.** The original gel image of EMSA.

## Data Availability

All data analysed in this study are included in this published article and its supplementary files. The sequences of the genes used and analysed in current study are available at Phytozome database (https://phytozome.jgi.doe.gov/pz/portal.html) and the accession numbers are as follows: ciclev10021695m, orange1.1g024441m, AT4G12350.1, GRMZM2G104551_T01, Potri.003G114100.1, Manes.02G034300.1, Thecc1EG016054t1, Gorai.003G168100.1, MDP0000682032, Glyma.01G211500.1, GSVIVT01004317001, mrna32268.1-v1.0-hybrid, Medtr4g102380.1, evm.model.supercontig_3.300, ciclev10028537m and ciclev10010416m. The plant materials are available from the corresponding author upon reasonable request.

## Abbreviations

IPP: Isopentenyl diphosphate;
DMAPP: Dimethylallyl diphosphate;
MVA: Mevalonate
MEP: Methylerythritol phosphate
FPP: Farnesyl diphosphate
SQS: Squalene synthase
SQE: Squalene epoxidase
OSC: Oxidosqualene cyclases
TF: Transcription factor
CDS: Coding sequence
ORF: Open reading frame
JC: Beibei 447 orange
NHE: Newhall navel orange
GXSTY: Guangxi Shatian pummelo
ZSXLMY: Early Siam pummelo
ST: Morita Unshu Mandarin
GC: Miyagawa Wase Mandarin
WCZPG: Wangcang Zoupigan mandarin
HPJG: Huapi kumquat
RAJG: Rongan kumquat
SD: Synthetic dropout
Ura: Uracil
Leu: Leucine
AbA: Aureobasidin A
EMSA: Electronic mobility shift assay.
