# Correction to: The MYB transcription factor CiMYB42 regulates limonoids biosynthesis in citrus

**DOI:** 10.1186/s12870-020-02491-4

**Published:** 2020-07-06

**Authors:** Pan Zhang, Xiaofeng Liu, Xin Yu, Fusheng Wang, Junhong Long, Wanxia Shen, Dong Jiang, Xiaochun Zhao

**Affiliations:** 1 Citrus Research Institute, Southwest University/Chinese Academy of Agricultural Sciences, Beibei, Chongqing, 400712 China; 2 National Citrus Engineering Research Center, Beibei, Chongqing, 400712 China

**Correction to: BMC Plant Biol 20, 254 (2020)**

**https://doi.org/10.1186/s12870-020-02475-4**

After publication of this article [1], it is reported that the name of the 3rd author is incorrect.

It should be ‘Xin Yu’ rather than ‘Xin Xu’.

This has been verified and the name has therefore been corrected in this erratum and been updated in the original article.
