# Supplementary material for: The MYB transcription factor CiMYB42 regulates limonoids biosynthesis in citrus
Source: BMC Plant Biol. 2020 Jun 3;20:254. doi: 10.1186/s12870-020-02475-4 (PMC7271526; doi:10.1186/s12870-020-02475-4)
Supplement: Supplementary file 1 — Additional file 1: Table S1. Primers and probes used in this study. [file 12870_2020_2475_MOESM1_ESM.docx]

Table S1. Primers and probes used in this study

| Primer name | Primer sequence | Description |
| --- | --- | --- |
| β-actin F | CCAAGCAGCATGAAGATCAA | The primers were used for normalization in quantitative analysis |
| β-actin R | ATCTGCTGGAAGGTGCTGAG |  |
| MYB42-F | GGGCGAACCGATAACGAGAT | The primers were used for quantitative analysis of *CiMYB42*. |
| MYB42-R | GCTGGAGTCTGGCAGCAAAT |  |
| SQS-F | TCCAGAAGTTTCGCTCTCGTTA | The primers were used for quantitative analysis of *CiSQS*. |
| SQS-R | TGGTGTCATCCTCAACAGTGTC |  |
| OSC-F | CTTTTGGCCTGGTGACTATGGT | The primers were used for quantitative analysis of *CiOSC*. |
| OSC-R | CATTTCCCTTCGATGTTCCACT |  |
| MYB42-OE-F | **GGACTCTAGAGGATC**ATGGGCAGGCAACCTTGC | The primers were used to generate the PBI121CSH-CiMYB42 overexpression vector. |
| MYB42-OE-R | **GATCGGGGAAATTCG**CTAGTGCCTGCCACCCAC |  |
| MYB42-1F | **ACGCTCGAGTATAAG**AAGCTCCTTAAAATGGGAATCGATCC | The primers were used to construct the first strand of pGBi-CiMYB42 RNAi vector. |
| MYB42-1R | **TTGTTGTAAAAATAG**CTAGTGCCTGCCACCCAC |  |
| MYB42-2F | **GCAGGTATTTGGATC**CTAGTGCCTGCCACCCAC | The primers were used to construct the reverse complement strand of pGBi-CiMYB42 RNAi vector. |
| MYB42-2R | **TAATTAACTCTCTAG**AAGCTCCTTAAAATGGGAATCGATCC |  |
| SQS-DF | **AAAAGCTTGAATTCG**GGATCCGATACGTCACTGG | The primers were used to construct the bait vector of pAbAi-SQS. |
| SQS-DR | **GAGCACATGCCTCGA**GGACTTGAGGAGAGAGGC |  |
| OSC-DF | **AAAAGCTTGAATTCG**TATCATATGTTGCAACGCAG | The primers were used to construct the bait vector of pAbAi-OSC. |
| OSC-DR | **GAGCACATGCCTCGA**GCTATATATATGAAGAGATTTGGAG |  |
| MYB42-DF | **CGAGCTCGATGGATC**ATGGGCAGGCAACCTTGC | The primers were used to construct the prey vector of pGADT7-MYB42. |
| MYB42-DR | **GGAGGCCAGTGAATT**CTAGTGCCTGCCACCCACC |  |
| OSC-1 | TGCTATAGTAT**ACCAAAC**ACGAGATAATA (-914to -885) | The probe was used to conduct the EMSA. |
| OSC-2 | ACTAGCGTCAACAAA**TTGTTG**AATTTAAA (-1207 to -1178) | The probe was used to conduct the EMSA. |
| OSC-3 | GTAATTGTAGAA**TAACTA**CGCTATGAAATA (-1754 to -1724) | The probe was used to conduct the EMSA. |
| MYB42-PF | **AGGGAAGGATTTCAGAATTC**GGATCCATGGGCAGGCAACCTTGCTG | The primers were used to construct pMAL-C2X-MYB42 vector with 6×His tag. |
| MYB42-PR | **ACGGCCAGTGCCAAGCTTGCCTGCAG**CTAATGGTGATGGTGATGGTG |  |

Note: The bold bases represent the extensions homologous to vector ends or the core sequence of *cis-element*.
