# Supplementary material for: The MYB transcription factor CiMYB42 regulates limonoids biosynthesis in citrus
Source: BMC Plant Biol. 2020 Jun 3;20:254. doi: 10.1186/s12870-020-02475-4 (PMC7271526; doi:10.1186/s12870-020-02475-4)
Supplement: Supplementary file 2 — Additional file 2: Figure S1. Schematic diagram of limonoid biosynthesis. The abbreviations of the compounds and enzymes are as follows: IPP, isopentenyl diphosphate; DMAPP, dimethylallyl diphosphate; FPP, farnesyl diphosphate; FPPS, FPP synthase; SQS, squalene synthase; SQE, squalene epoxidase, OSC, oxidosuqlene cyclase; UDPG, UDP-glycosyltransferase; NG, nomilin-glucopyranoside; LG, limonin-glucopyranoside. [file 12870_2020_2475_MOESM2_ESM.docx]

Squalene

SQS

DMAPP

IPP

2IPP+DMAPP

FPP (C15)

FPPS

2, 3-oxidosqualene

SQE

Nomilin

OSC

LG

NG

Limonin

UDPG

UDPG

**MVA pathway**

**MEP pathway**

Figure S1. Schematic diagram of limonoid biosynthesis. The abbreviations of the compounds and enzymes are as follows: IPP, isopentenyl diphosphate; DMAPP, dimethylallyl diphosphate; FPP, farnesyl diphosphate; FPPS, FPP synthase; SQS, squalene synthase; SQE, squelene epoxidase, OSC, oxidosuqlene cyclase; UDPG, UDP-glycosyltransferase; NG, nomilin-glucopyranoside; LG, limonin-glucopyranoside.
