# Supplementary material for: The MYB transcription factor CiMYB42 regulates limonoids biosynthesis in citrus
Source: BMC Plant Biol. 2020 Jun 3;20:254. doi: 10.1186/s12870-020-02475-4 (PMC7271526; doi:10.1186/s12870-020-02475-4)
Supplement: Supplementary file 3 — Additional file 3: Figure S2. Developmental stages of the leaf samples used in this study. [file 12870_2020_2475_MOESM3_ESM.docx]

Figure S2. Developmental stages of the leaf samples used in this study
