# Supplementary material for: The MYB transcription factor CiMYB42 regulates limonoids biosynthesis in citrus
Source: BMC Plant Biol. 2020 Jun 3;20:254. doi: 10.1186/s12870-020-02475-4 (PMC7271526; doi:10.1186/s12870-020-02475-4)
Supplement: Supplementary file 4 — Additional file 4: Figure S3. The TF-binding cis-elements of the CiSQS and CiOSC promoters. [file 12870_2020_2475_MOESM4_ESM.docx]

Figure S3. The TF-binding *cis*-elements of the *CiSQS* and *CiOSC* promoters.
