# Supplementary material for: The MYB transcription factor CiMYB42 regulates limonoids biosynthesis in citrus
Source: BMC Plant Biol. 2020 Jun 3;20:254. doi: 10.1186/s12870-020-02475-4 (PMC7271526; doi:10.1186/s12870-020-02475-4)
Supplement: Supplementary file 5 — Additional file 5: Figure S4. The minimal AbA inhibitory concentration of the bait vector. (a) pAbAi-SQS; (b) pAbAi-OSC. [file 12870_2020_2475_MOESM5_ESM.docx]

Figure S4. The minimal AbA inhibitory concentration of the bait vector; (a) pAbAi-SQS; (b) pAbAi-OSC.


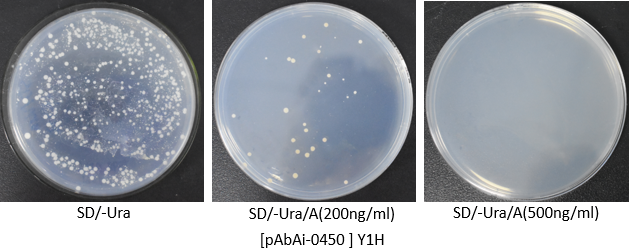


SD/-Ura

SD/-Ura/A (200ng/mL)

SD/-Ura/A (500ng/mL)


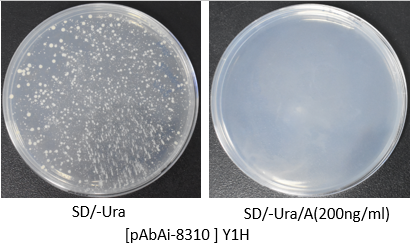


SD/-Ura

SD/-Ura/A (200ng/mL)

**a**

**b**
