# Supplementary material for: The MYB transcription factor CiMYB42 regulates limonoids biosynthesis in citrus
Source: BMC Plant Biol. 2020 Jun 3;20:254. doi: 10.1186/s12870-020-02475-4 (PMC7271526; doi:10.1186/s12870-020-02475-4)
Supplement: Supplementary file 6 — Additional file 6: Figure S5. Expression and purification of the CiMYB42 protein. (a) The recombinant CiMYB42 protein expressed in Rosetta (DE3); (b) The supernatant and deposition of the CiMYB42 protein were examined by SDS-PAGE after ultrasonication; (c) SDS-PAGE analysis of recombinant and purified CiMYB42 protein; Lane M: protein ladder (116.0/66.2/45.0/35.0/25.0/18.4/14.4 kD); Lane 1: uninduced Rosetta (DE3) carrying pMAL-C2X; Lane 2: Rosetta (DE3) carrying pMAL-C2X induced by 1 mM IPTG; Lane 3: supernatant of the CiMYB42 protein; Lane 4: precipitate of the CiMYB42 protein; Lane 5: purified CiMYB42 protein; Lane 6: purified CiMYB42 protein. The molecular weight of recombinant CiMYB42 protein is 72.21kD. The gel image were cropped because original image includes some of other samples not related to this study. [file 12870_2020_2475_MOESM6_ESM.docx]

Figure S5. Expression and purification of the CiMYB42 protein.

(a) The recombinant CiMYB42 protein expressed in Rosetta (DE3); (b) The supernatant and deposition of the CiMYB42 protein were examined by SDS-PAGE after ultrasonication; (c) SDS-PAGE analysis of recombinant and purified CiMYB42 protein;

Lane M: protein ladder (116.0/66.2/45.0/35.0/25.0/18.4/14.4 kD);

Lane 1: uninduced Rosetta (DE3) carrying pMAL-C2X;

Lane 2: Rosetta (DE3) carrying pMAL-C2X induced by 1mM IPTG;

Lane 3: supernatant of the CiMYB42 protein;

Lane 4: precipitate of the CiMYB42 protein;

Lane 5: purified CiMYB42 protein;

Lane 6: purified CiMYB42 protein.

The molecular weight of recombinant CiMYB42 protein is 72.21kD. The gel image were cropped because original image includes some of other samples not related to this study.
