# Supplementary material for: The MYB transcription factor CiMYB42 regulates limonoids biosynthesis in citrus
Source: BMC Plant Biol. 2020 Jun 3;20:254. doi: 10.1186/s12870-020-02475-4 (PMC7271526; doi:10.1186/s12870-020-02475-4)
Supplement: Supplementary file 7 — Additional file 7 Figure S6. The original gel image of Figure S5. (a) The original image of Figure S5a; (b) The original image of Figure S5b; (c) The original image of Figure S5c. [file 12870_2020_2475_MOESM7_ESM.docx]

a


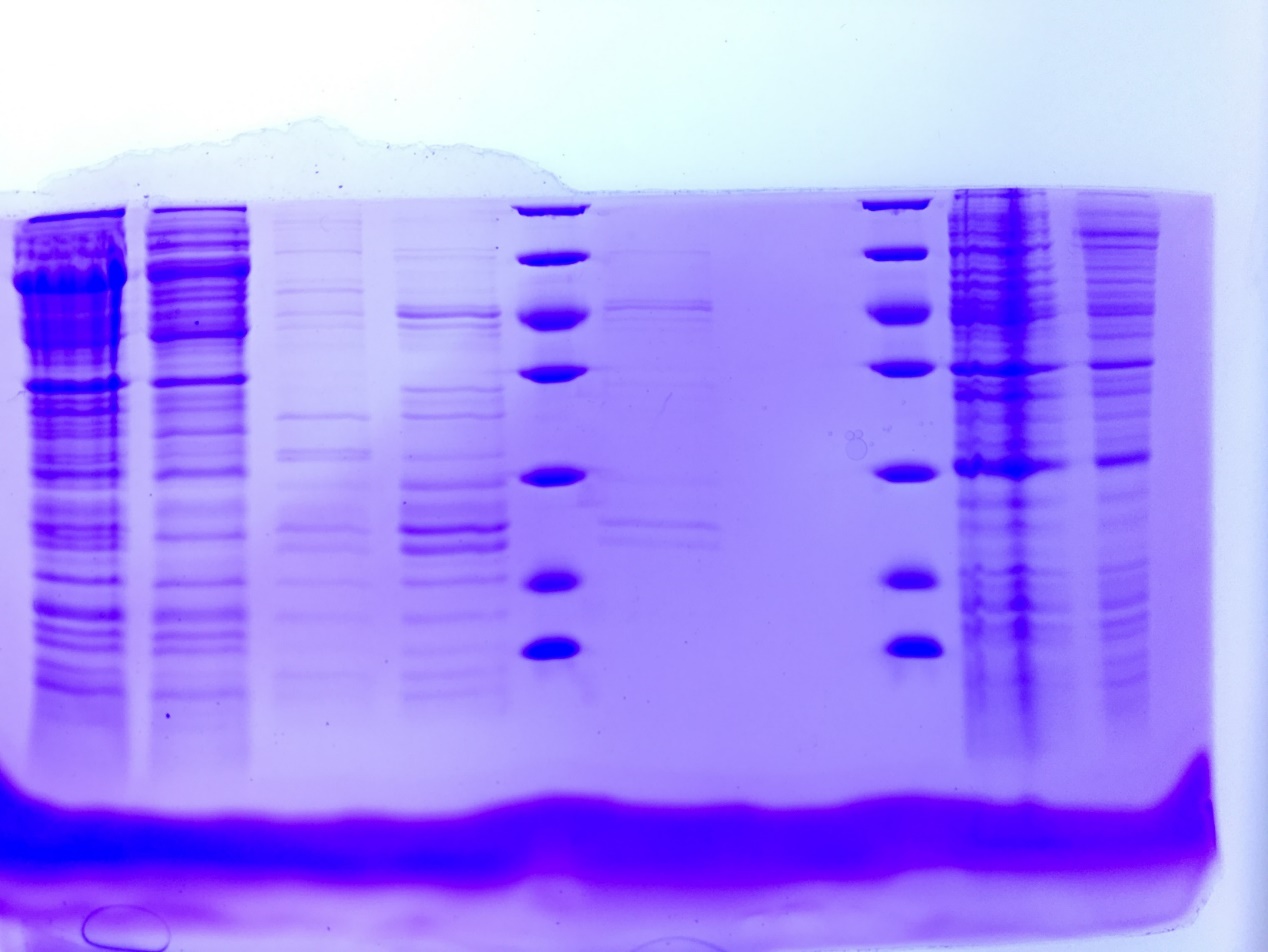


b


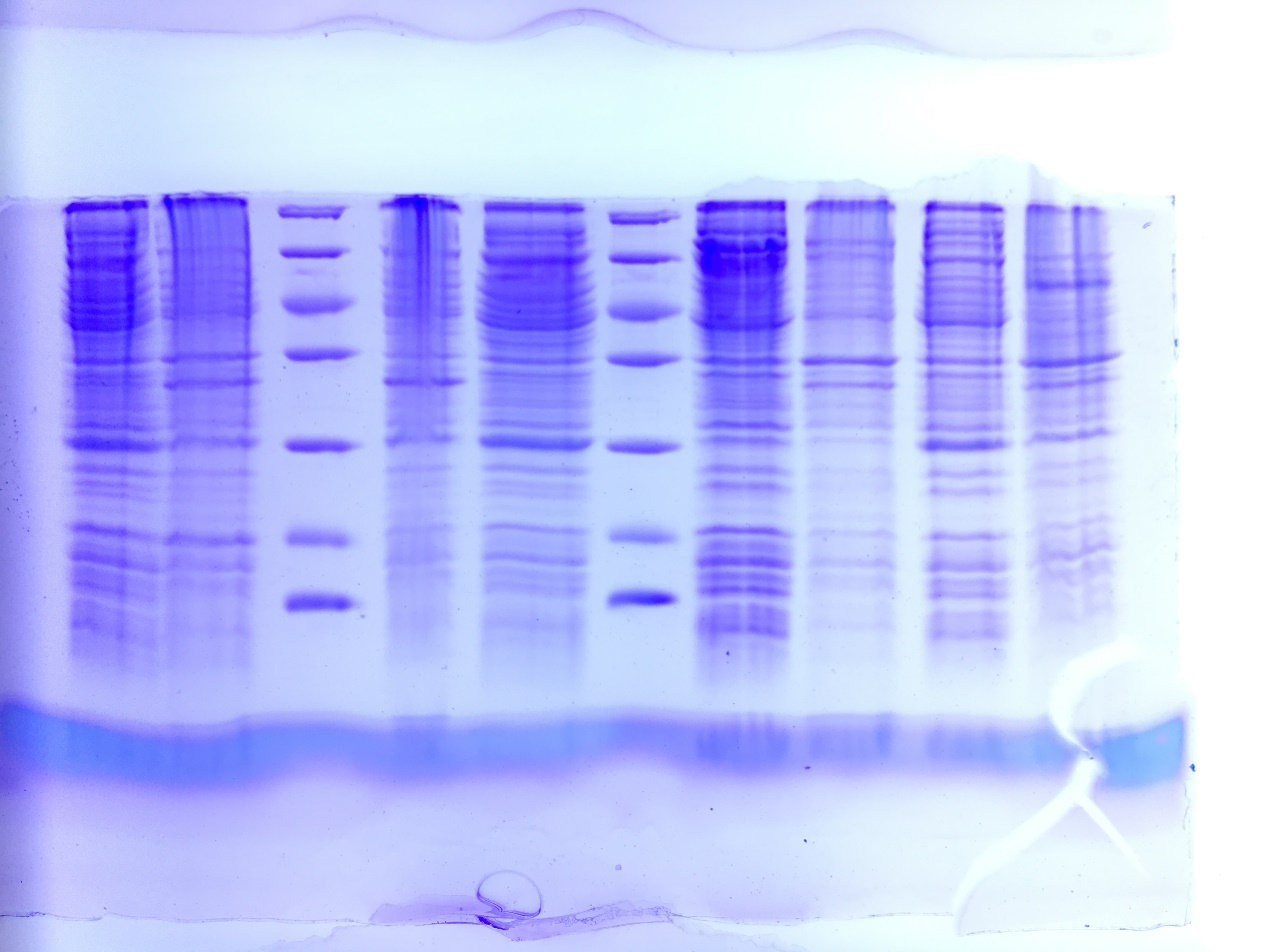


c


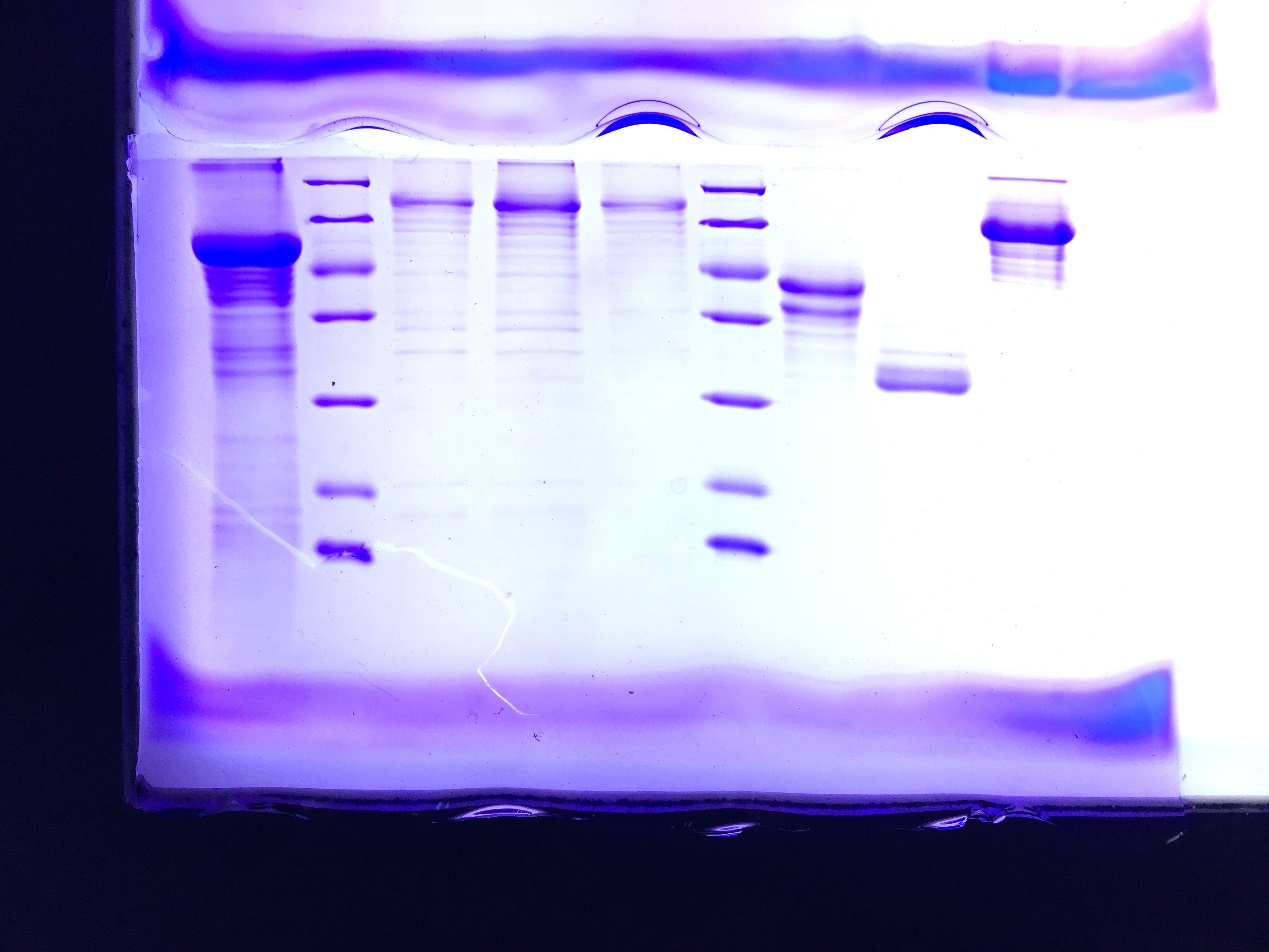


Figure S6 The original gel image of Figure S5. (a) The original image of Figure S5a; (b) The original image of Figure S5b; (c) The original image of Figure S5c.
